# Movement kinematics and cortical activation in children with and without autism spectrum disorder during sway synchrony tasks: an fNIRS study

**DOI:** 10.1038/s41598-021-94519-4

**Published:** 2021-07-22

**Authors:** Wan-Chun Su, McKenzie Culotta, Daisuke Tsuzuki, Anjana Bhat

**Affiliations:** 1 Department of Physical Therapy, University of Delaware, 540 South College Avenue, Newark, DE USA; 2 Biomechanics and Movement Science Program, University of Delaware, Newark, DE USA; 3 Department of Language Sciences, Tokyo Metropolitan University, Tokyo, Japan; 4 Department of Psychological and Brain Sciences, University of Delaware, Newark, DE USA

**Keywords:** Social neuroscience, Biomarkers

## Abstract

Children with Autism Spectrum Disorder (ASD) have difficulties with socially embedded movements such as imitation and interpersonal synchrony (IPS); however, related movement characteristics and underlying neural mechanisms are not well understood. This study compared the movement characteristics and cortical activation patterns of children with and without ASD during a whole-body, sway synchrony task when different levels of social information were provided. Thirty children with and without ASD (mean age: 12.6 years, SE: 0.6 years) participated. Movement kinematics and fNIRS-based cortical activation were recorded when the child observed an adult tester sway side to side, when they swayed solo, or when they swayed face to face with the tester with or without fingertips touching (i.e., IPS). Children with ASD showed reduced synchrony and smaller sway amplitude compared to typically developing children without ASD. They showed reduced cortical activation over the inferior frontal gyrus and superior temporal sulcus during IPS and did not show significant increase in cortical activation when more social information was provided. The cortical activation findings were significantly associated with IPS behaviors and social communication performance. The ASD-related neurobiomarkers identified in our study could be used as objective measures to evaluate intervention effects in children with ASD.

## Introduction

Autism Spectrum Disorder (ASD) is a highly prevalent neurodevelopmental disorder affecting 1 in 59 children in the US^1^. Children with ASD present with diagnostic symptoms of impaired social communication skills (DSM-5)^2^ as well as perceptuo-motor comorbidities which affects their ability to move with others within social contexts^3^. For example, children with ASD might have impaired social monitoring and poor motor planning/incoordination that in turn affects their ability to imitate (i.e., copy discrete actions)^4^ or synchronize continuous actions with others, also known as interpersonal synchrony (IPS)^5,6^. During infancy and early childhood, infants and children learn a variety of social communication skills from their social partners by engaging in imitation and IPS^7,8^. These interactions help facilitate children’s relationships with their caregivers and peers^9,10^. Difficulties in engaging in imitation and IPS will affect the social cognitive development of children with ASD and will limit their opportunities to build relationships with other social partners^11^.

Studies have reported reduced IPS and atypical movement characteristics including slower movement speed and inaccurate movements in children with ASD^5,12^. The neuroimaging studies also suggested atypical activation over the frontal, temporal and parietal lobes when children with ASD performed imitation and IPS tasks^13,14^. However, the neuroimaging studies were limited to finger motions or seated reaching tasks that did not involve whole-body movements or use of objective kinematic measures using motion tracking. Moreover, changing levels of social information (e.g., social-visual or social-tactile) could variably influence IPS behaviors and corresponding cortical activation. Using functional near-infrared spectroscopy (fNIRS), a novel neuroimaging tool that is robust against movement artifacts, the present study compared the movement characteristics and associated cortical activation in children with and without ASD during a whole-body sway synchrony task and investigated how different levels of social information might alter IPS behaviors and associated cortical activation.

### IPS behaviors and associated cortical activation

IPS is an important everyday activity that requires complex social and perceptuo-motor skills. Some example of IPS include people walking at the same pace or dancing with a partner^15,16^. When engaging in IPS, one needs to understand the shared goal of the task as well as one’s own individual role in the task^17^. For example, when dancing with a partner, the common goal is to move in harmony, while the individual goals might focus on moving limbs and shifting weight in a certain direction. Additionally, partners need to perceive and monitor the information from the environment (e.g., the locations of the obstacles in the room), and based on their own and their partner’s actions (e.g., eye gaze, body posture, etc.)^17^. The environmental and social information will help in anticipating/predicting the partner’s movements and reactively adjusting one’s own movements to keep up with those of the partner’s^17^. During synchronization, one might perceive faster or slower movements of their partner and plan ahead or reactively change the speed of their own movements to stay in synchrony with the partner. The monitoring and adjustments of actions during IPS requires continuous perceptuo-motor integration which is also needed during imitation and other joint actions^17^.

Several cortical regions in bilateral frontal, parietal, and temporo-occipital lobes that support imitation behaviors may also play a role in IPS^18^. The inferior frontal gyrus (IFG) plays a role in encoding the goal of actions as it is more active when performing goal-directed than non-goal directed movements^19^. It also plays a role in perceiving the salience of action information offered by partners and in inferring intentions of actions^20^. Furthermore, fronto-parietal connections are important in integrating multisensory information from the environment and social partners^21^. The IFG and inferior parietal lobe (IPL) interact with each other and are important for matching observed actions with internal motor plans and anticipatory/predictive control of actions^22,23^. The superior temporal sulcus (STS) plays an important role in establishing visuomotor correspondence and is activated as one monitors and matches their own actions to those of their partner’s in a predictive or reactive manner^24^. In fact, previous fMRI studies found that IFG, STS, and IPL regions are important parts of the imitation network and are more active during imitation compared to its component behaviors, including action observation and execution^25^. Other important brain regions for IPS include pre- and post-central gyrus (PCG) and the prefrontal regions (including middle frontal gyrus (MFG)). PCG includes the primary motor and somatosensory cortices that interact with other cortical regions to receive/process sensory information and execute actions^26^. The MFG along with other prefrontal regions are important for executive functions such as motor planning, working memory, cognitive shifting, and inhibition—a set of mental skills that are important during continuous actions involving IPS^27^. During IPS, one needs to engage the aforementioned cortical regions to retain information about the goal/task requirements and the spatial relationships between oneself and the partner, plan one’s actions, shift one’s attention to critical components of the actions, and inhibit irrelevant movements to match up with the partner’s actions. These cortical regions not only interact with each other, but also with other cortical (e.g., occipital cortices) and subcortical regions (e.g., cerebellum is important for predictive control)^28,29^.

### IPS difficulties in children with ASD

Children and adolescents with ASD show reduced IPS during joint pendulum swaying and synchronized marching and clapping^5,6^. They also showed atypical movement characteristics during IPS (i.e., reduced temporal accuracy and altered movement speed) suggesting impaired social perception and solo coordination of actions^5,12^. More specifically, impairments in goal understanding, social monitoring, executive functioning, as well as poor anticipatory and reactive control of movements in children with ASD might contribute to their difficulties in engaging in imitation and IPS tasks with others^30–33^.

### Atypical cortical activation during IPS in children with ASD

Besides the behavioral findings, children with ASD also show atypical cortical activation during imitation/IPS and its component behaviors (i.e., action observation and execution). Multiple fMRI studies have reported atypical activation over frontoparietal and superior temporal regions in children with ASD when imitating finger movements and hand gestures^34,35^. While some electroencephalography research fails to duplicate the findings of atypical activation^36^, a thorough meta-analysis of imitation task data from fMRI studies found that individuals with ASD had increased IPL activation and altered activation over the occipital, dorsolateral prefrontal, and cingulate cortices, as well as the insula, compared to control participants^14^.

### functional Near-infrared spectroscopy (fNIRS)

Although fMRI studies have identified possible neurobiomarkers of atypical imitation in individuals with ASD, the tasks were limited to simple finger and hand movements due to the constraints of the fMRI scanning bore. Functional near-infrared spectroscopy (fNIRS) is a non-invasive neuroimaging technique that constrains the subject only by a cap, allows upright face to face interactions, and is robust against movement artifacts^37^, making it a favorable approach to implement in children with ASD. Using fNIRS, our research group has reported atypical cortical activation, i.e., reduced IFG and STS activation, and increased IPL activation in children with ASD during a face to face reaching synchrony task^13^.

### Aims and hypotheses

In the present study, we extended our past research to a whole-body, sway synchrony task, expanded the fNIRS probe set to better cover the prefrontal cortices, used motion tracking to objectively assess body movement characteristics, and manipulated social information during the sway synchrony task. We hypothesize that the children with ASD will have atypical IPS and sway amplitude compared to their TD peers. They will also have atypical frontoparietal and temporal activation during sway synchrony compared to TD peers. Moreover, we hypothesize that the sway characteristics and cortical activation of children with ASD will correlate with each other and with their social communication performance.

## Results

### Movement characteristics during IPS

**Figure 1:**
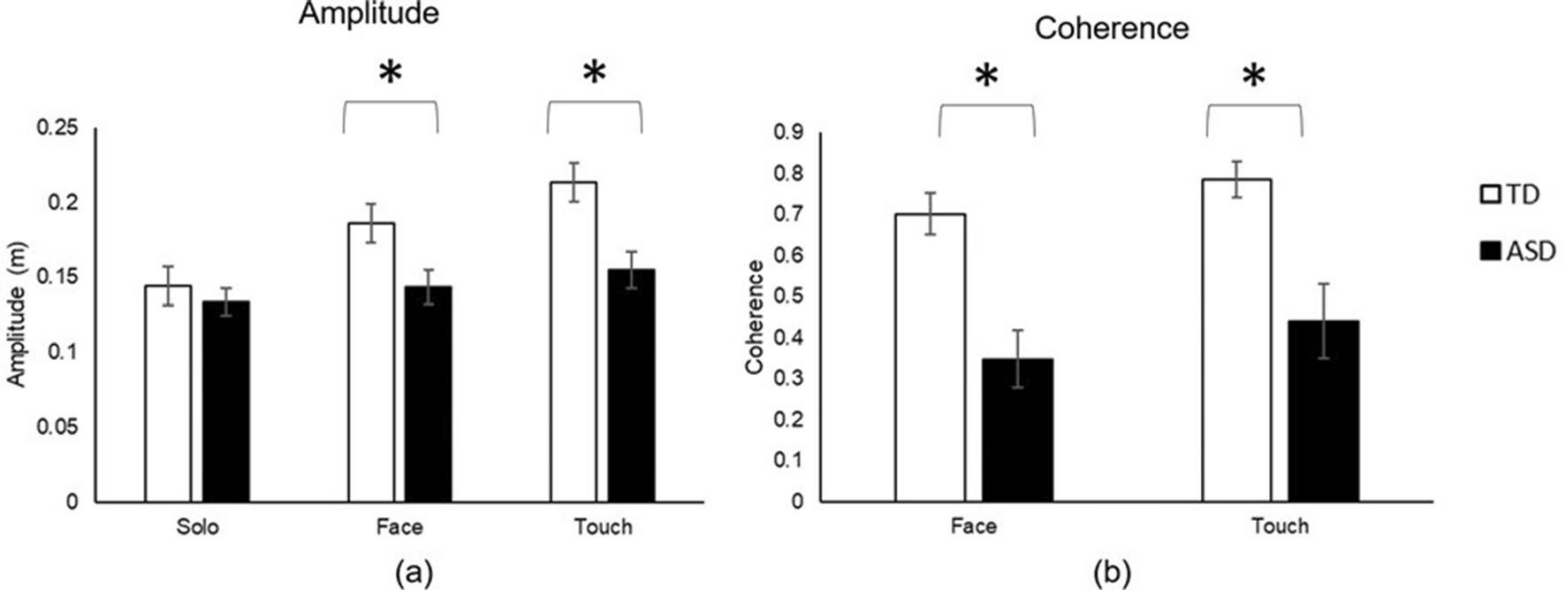
Movement amplitude (**a**) and coherence (**b**) during interpersonal synchrony. *Indicates significant differences between the ASD and TD groups.

Movement characteristics differed between children with and without ASD in the Social (Face and Touch) but not Solo conditions. Specifically, both groups showed similar sway amplitude in the Solo (*p* > 0.05) but children with ASD showed reduced sway amplitude in the Face and Touch conditions compared to the TD children (*p*s < 0.05, Hedge’s g = 0.05 (95% CI = − 0.67 ~ 0.77) & 0.07 (95% CI = − 0.65 ~ 0.78); Fig. 1a). For sway coherence with the tester, children with ASD showed significantly lower coherence compared to the TD children in both Face and Touch conditions (*p*s < 0.01, Hedge’s g = 0.20 (95% CI = − 0.52 ~ 0.92) & 0.21 (95% CI = − 0.51 ~ 0.93); Fig. 1b), indicating poor IPS in children with ASD.

### Cortical activation during IPS

**Figure 2:**
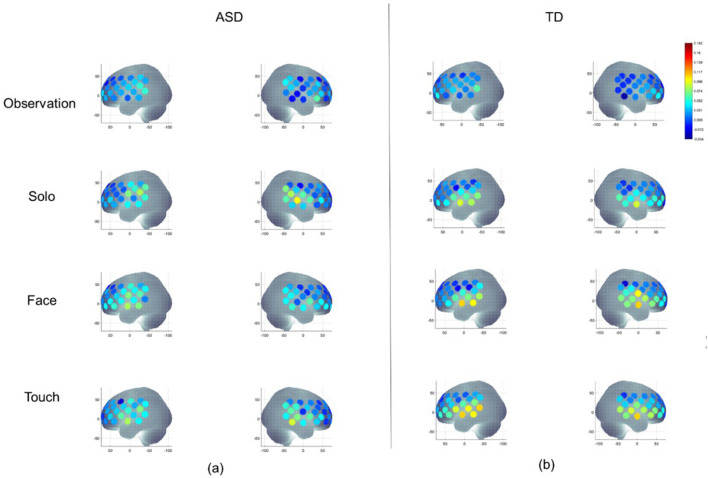
A visual representation of averaged HbO_2_ concentration during Observation, Solo, Face, and Touch conditions in children with ASD (**a**) and the TD children (**b**). HbO_2_ values on Y-axis range from 0 indicated by blue to 0.18 indicated by red. In Observation and Solo conditions, children with ASD had greater IPL activation than the TD children. During Face condition, children with ASD had lower STS activation than the TD children. Similarly, in Touch condition, children with ASD had lower IFG and STS activation than the TD children.

The Group × Condition × Hemisphere × Region four-way repeated ANOVA revealed a significant main effect of Hemisphere (F(1.0, 115.0) = 6.034, *p* = 0.016), a 2-way interaction of Condition × Region (F(8.3, 955.1) = 2.5, *p* < 0.05), and a 3-way interaction of Group × Condition × Region (F(8.3, 955.1) = 1.9, *p* = 0.05). The Group × Condition × Region interaction did not co-vary with the sway amplitude or BOT-2 Body coordination scores, therefore, post-hoc analyses were done to further explore this interaction. The visual representation of averaged HbO_2_ concentration during all four conditions in both groups is shown in Fig. 2. The means and standard errors (SE) of HbO_2_ concentrations are presented in Supplementary Table S1 and the results of post hoc analyses are presented in Supplementary Table S2 online.

### Group differences

**Figure 3:**
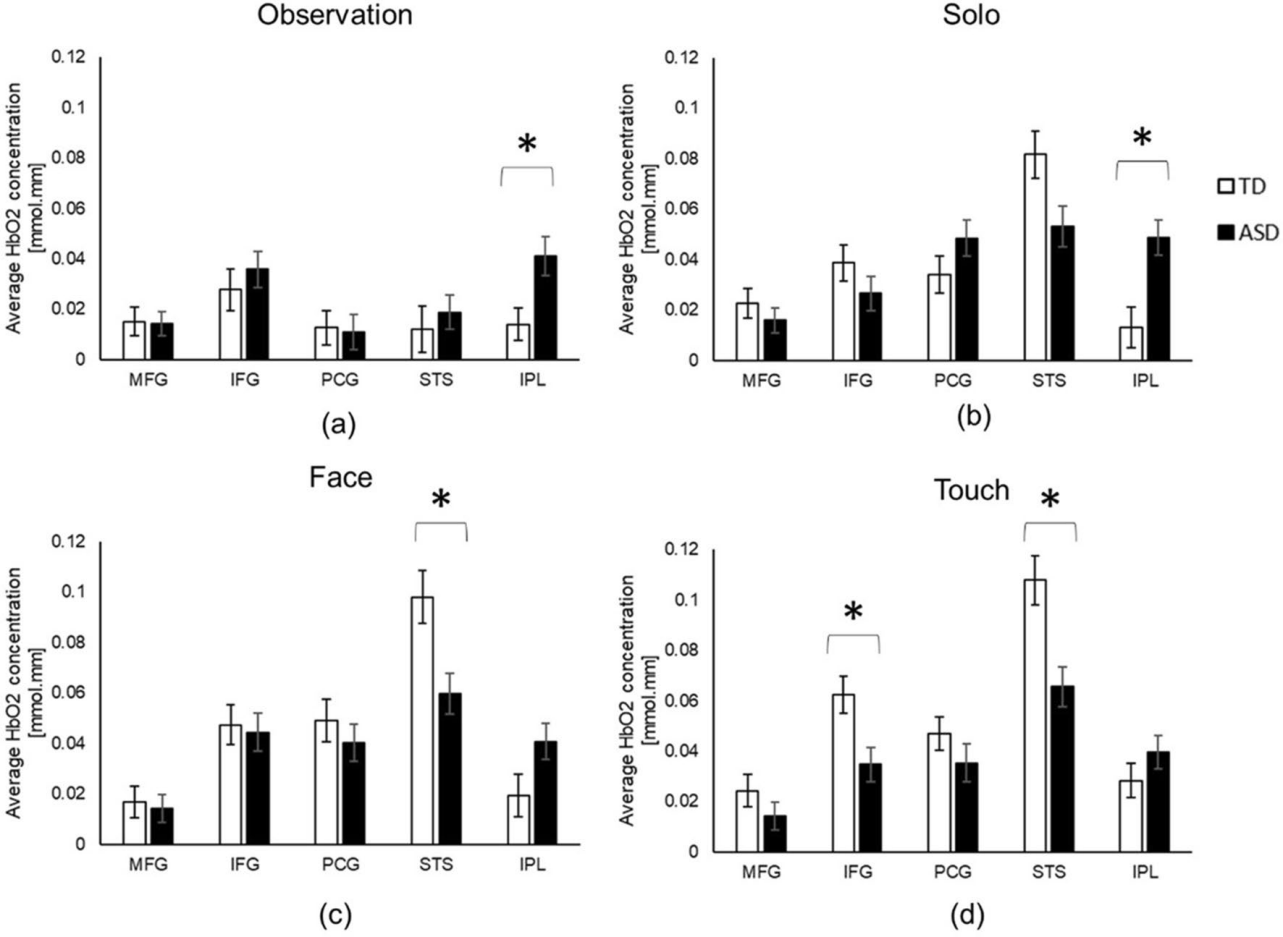
Group differences in HbO_2_ concentration during Observation (**a**), Solo (**b**), Face (**c**), and Touch (**d**) conditions. *Indicates significant differences between the ASD and TD groups.

During Observation and Solo conditions, children with ASD showed greater IPL activation than TD children (*p*s < 0.01, Hedges g = 0.04 (95% CI = − 0.68 ~ 0.76) & 0.06 (95% CI = − 0.66 ~ 0.77); Fig. 3a,b). During the Face condition, children with ASD showed lower STS activation than the TD children (*p* < 0.01, Hedges g = 0.06 (95% CI = − 0.77 ~ 0.66); Fig. 3c). Similarly, in the Touch condition, children with ASD had lower IFG and STS activation than the TD children (*p*s < 0.01, Hedges g = 0.04 (95% CI = − 0.77 ~ 0.67) & 0.06 (95% CI = − 0.75 ~ 0.68); Fig. 3d).

### Condition-related differences

**Figure 4:**
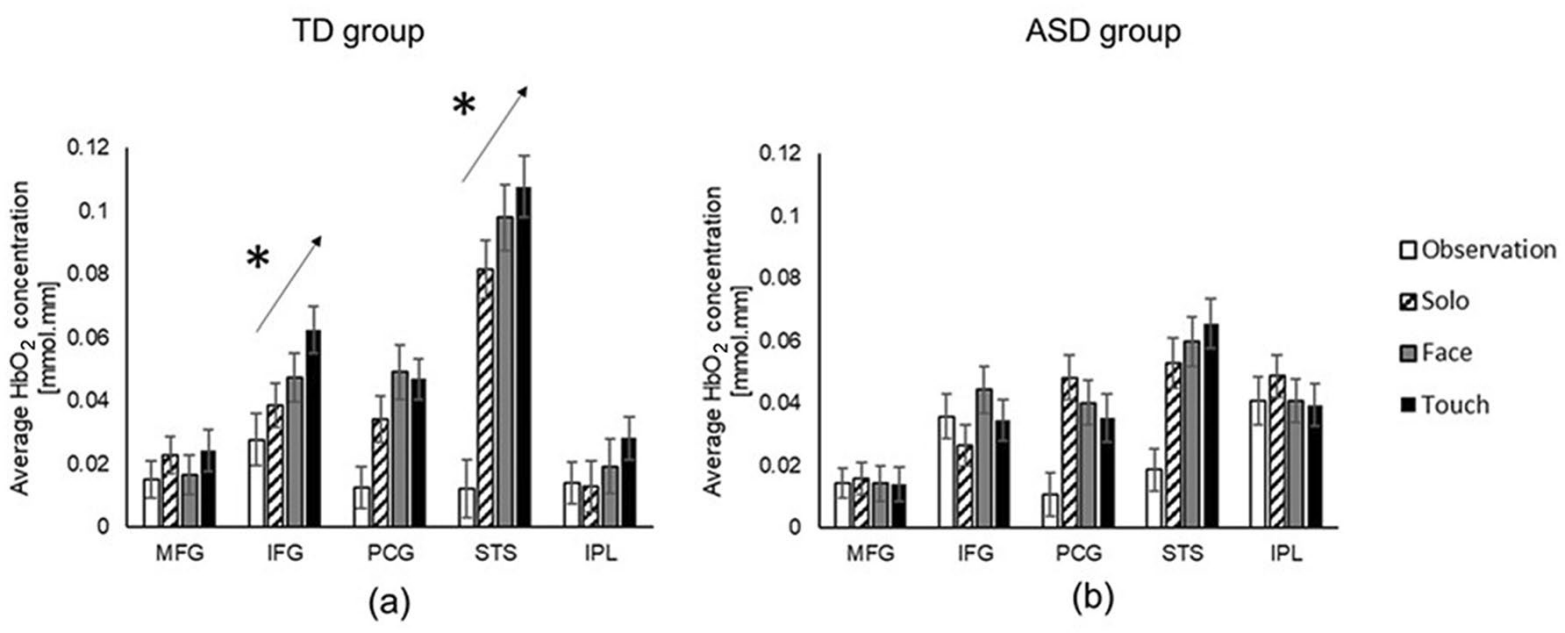
Conditional differences in HbO_2_ concentration for the TD children (**a**) and children with ASD (**b**). *Indicate significant differences between movement conditions.

Both groups showed greater PCG and STS activation during the movement conditions (Solo, Face, and Touch) compared to the Observation condition (*p*s < 0.05, Hedge’s g = 0.05 ~ 0.13 (95% CI = − 0.83 ~ 0.67); Figs. 4a and 4b). In the movement conditions, the TD children scaled up their IFG and STS activation when more social information was available (IFG: Touch > Face and Solo, *p*s < 0.05; PCG: Touch > Solo, *p*s < 0.05, Hedge’s g = 0.03 ~ 0.04 (95% CI = − 0.77 ~ 0.68); Fig. 4a). However, children with ASD had no significant differences between movement conditions (Solo ≈ Face ≈ Touch, *p*s > 0.05, Fig. 4b).

### Correlation between IPS behaviors and cortical activation

In the TD children, greater sway amplitude was associated with greater right PCG and IPL activation during the Face condition (right PCG: r = 0.46, right IPL: r = 0.41, *p*s < 0.001) whereas in the children with ASD, greater sway amplitude was associated with greater left STS and right PCG activation during the Touch condition (left STS: r = 0.48, right PCG: r = 0.42, *p*s < 0.001). In the TD children, greater sway coherence was associated with greater right PCG activation during the Face condition (r = 0.45, *p* < 0.001); however, there was no significant correlation between coherence and cortical activation in the children with ASD (Table 1).

**Table 1:** Correlations between cortical activation and interpersonal synchrony behaviors.

r-values	Sway amplitude		Sway coherence	
	Face	Touch	Face	Touch
**TD**				
Left hemisphere				
MFG	0.105	0.098	0.229	0.262*
IFG	0.213	0.141	0.263*	0.179
PCG	0.210	0.211	0.287*	− 0.019
STS	0.236	0.287*	0.292*	− 0.151
IPL	0.087	0.129	0.076	0.018
Right hemisphere				
MFG	0.212	0.117	0.249	0.267*
IFG	0.326*	0.332*	0.329*	0.374**
PCG	0.463**	0.247	0.445**	0.148
STS	0.286*	0.087	0.354**	− 0.075
IPL	0.410**	0.141	0.299*	− 0.188
**ASD**				
Left hemisphere				
MFG	0.122	0.086	− 0.078	− 0.355**
IFG	0.131	0.288*	− 0.266*	− 0.075
PCG	0.111	0.369**	− 0.231	− 0.299*
STS	0.346**	0.482**	0.069	− 0.156
IPL	0.190	0.327*	− 0.148	− 0.074
Right hemisphere				
MFG	0.159	0.204	− 0.210	− 0.189
IFG	0.194	0.222	− 0.191	− 0.043
PCG	0.356**	0.423**	− 0.104	− 0.180
STS	0.258*	0.233	− 0.327*	− 0.290*
IPL	0.003	0.303*	0.019	0.015

Bolded font indicates *p* values survived after FDR corrections. r values are presented in this figure.

*Indicates *p* < 0.05.

**Indicates *p* < 0.01.

### Correlation between social communication questionnaires and cortical activation

Only two correlations were noted in the TD group: Greater VABS communication scores were associated with greater left MFG activation during the Face condition (r = 0.37, *p* < 0.001) whereas greater VABS socialization scores were associated with lower right IFG activation during the Touch condition (r = − 0.43, *p* < 0.001). In the ASD group, better VABS communication performance was associated with greater right PCG activation during the Face condition (r = 0.41, *p* < 0.001), and greater left STS, left IPL, and right PCG activation during the Touch condition (rs > 0.45, *p*s < 0.001). Furthermore, better VABS socialization performance was associated with greater right PCG activation (r = 0.45, *p* < 0.001), better SRS performance (i.e., lower SRS scores) was associated with greater left STS and right PCG activation (rs < − 0.35, *p* < 0.001), and greater ICS score was associated with greater left IFG activation during the Touch condition (r = 0.44, *p* < 0.001, Table 2).

**Table 2:** Correlations between cortical activation and social communication performance.

r-values	VABS communication		VABS socialization		SRS		ICS	
	Face	Touch	Face	Touch	Face	Touch	Face	Touch
**TD**								
Left hemisphere								
MFG	0.369**	0.229	0.04	− 0.037	− 0.068	− 0.111	0.079	0.245
IFG	0.217	0.161	− 0.051	− 0.116	− 0.162	− 0.247	0.109	0.279*
PCG	− 0.085	− 0.090	− 0.032	− 0.017	0.376**	0.128	− 0.358**	− 0.264*
STS	− 0.095	− 0.155	− 0.048	0.221	0.190	− 0.118	− 0.020	0.142
IPL	0.080	0.205	0.209	0.112	0.393**	0.143	− 0.115	− 0.084
MFG	0.286*	0.091	− 0.034	− 0.087	0.000	− 0.007	0.098	0.154
IFG	0.215	0.115	− 0.201	− 0.427*	− 0.144	− 0.257*	0.000	0.133
PCG	0.318*	0.095	− 0.332*	− 0.168	− 0.409**	− 0.221	0.236	0.230
STS	0.102	0.099	− 0.157	− 0.083	− 0.120	− 0.192	0.139	0.188
IPL	0.293*	0.082	− 0.290*	0.033	− 0.246*	0.005	− 0.014	0.267*
**ASD**								
Left hemisphere								
MFG	0.011	0.284*	0.113	0.205	− 0.069	− 0.162	0.219	0.186
IFG	− 0.152	0.163	− 0.094	0.098	0.158	0.008	0.101	0.436**
PCG	− 0.108	0.275*	0.048	0.198	0.184	− 0.153	0.205	0.184
STS	0.328**	0.543**	0.136	0.345**	− 0.246	− 0.376**	0.178	− 0.058
IPL	0.172	0.479**	0.261*	0.271*	− 0.066	− 0.323*	− 0.005	0.347**
Right hemisphere								
MFG	0.032	0.193	0.083	0.129	− 0.137	− 0.096	0.156	0.037
IFG	0.093	0.331**	0.208	0.287*	− 0.026	− 0.153	− 0.038	0.017
PCG	0.410**	0.555**	0.284*	0.454**	− 0.285*	− 0.354**	0.171	0.184
STS	0.303*	0.204	0.192	0.073	− 0.164	0.028	− 0.003	− 0.002
IPL	− 0.029	0.224	0.140	0.068	− 0.078	− 0.020	0.064	0.127

Bold font indicates *p* values survived for FDR corrections.

r values are presented in this table. *indicates *p* < 0.05; ** indicates *p* < 0.01.

## Discussion

Children with ASD have social and perceptuo-motor difficulties that affect their abilities to engage in IPS^6^. Previous fMRI and fNIRS studies have reported atypical cortical activation associated with imitation and IPS difficulties in adults and children with ASD^13,14^. However, the studies were limited to finger movements or seated reaching tasks without involving whole-body movements. The present study extended our past work studying reaching-related IPS to a whole-body sway synchrony task and investigated how different levels of social information might affect the IPS behaviors and cortical activation in children with and without ASD.

Consistent with past studies, children with ASD had reduced IPS and smaller sway amplitude compared to the TD children. In terms of cortical activation, children with ASD showed greater IPL activation during the Observation and Solo conditions, reduced STS activation during the Face condition, and reduced IFG and STS activation during the Touch condition. When more social information was provided (social-visual and/or social-visual and tactile), children with ASD did not show a significant increase in IFG and STS activation, unlike their TD peers. Cortical activation in children with ASD correlated with their movement characteristics during the sway synchrony task as well as their social communication performance.

Children with ASD showed reduced IPS and smaller sway amplitude compared to the TD children. The group differences in sway amplitude were only seen in the IPS conditions (Face and Touch) but not the Solo condition, indicating differences during socially embedded actions. Similar IPS difficulties have been reported in children with ASD during synchronized pendulum swaying^5^, marching and clapping^6^, and virtual tightrope walking tasks^54^. During the aforementioned tasks, children with ASD showed lower temporal accuracy, poor coordination, and greater movement variability^5,54^. The IPS difficulties of children with ASD might be rooted in their poor multi-limb and visuo-motor coordination, leading to a greater challenge during social synchrony^54,55^. Besides the motor components, children with ASD have social perceptual deficits (i.e., reduced social gaze, or poor perception of other’s actions) which could also undermine their ability to produce synchronized actions^56^. Together, these sensory-motor and social-perceptual difficulties may contribute to poor IPS performance in children with ASD.

We found greater IPL activation in children with ASD during Observation and Solo conditions compared to the TD children. Similar findings of hyperactivation over the IPL region in children with ASD have been reported in previous fMRI and fNIRS studies involving simple finger and hand movement imitation/IPS^13,14^. The IPL region is part of the default mode network (DMN)^57^, a network that is more active during internally directed processing (i.e., mind wandering and self-referencing), but is deactivated during externally directed processing (i.e., performing cognitively demanding tasks and goal-directed movements such as IPS)^58^. DMN is important in a wide range of emotions and social functions and share similar cortical substrates including regions important for imitation^59,60^. An fMRI study found more DMN deactivation when being imitated, compared to when individuals were imitating other’s movements, suggesting that DMN is more active when trying to match up to another individual’s actions^60^. Consistent with the previous finding, the TD children showed more deactivation during Observation and Solo conditions and less deactivation/more activation over the IPL regions during Face and Touch conditions. Children with ASD in general did not show deactivation of the IPL region during all conditions and had much greater overall IPL activation compared to TD children. It is possible that children with ASD were less focused on the externally-directed goals of solo/social synchrony and had reduced deactivation over the IPL region. Children with ASD are also known to have problems with deactivation during other cognitive tasks requiring visual inspection of complex patterns as well as working memory tasks^61,62^.

Consistent with the previous fMRI and fNIRS findings^13,14^, we found hypoactivation in IFG and STS in children with ASD during IPS. The IFG and STS activation was associated with the social communication and IPS performance in children with ASD. IFG is important for goal/intention understanding during action execution and imitation^19,28^. While observing object manipulation, the IFG was more active when the participants were told to focus on the goal but not the actions associated with the task^63^. Children with ASD have difficulties understanding goals or intentions underlying others’ actions^33^, and the difficulties have been linked to lower IFG activation during action observation^64^. During the sway synchrony task in this study, children with ASD might have focused more on their individual movements instead of the shared goal (i.e., moving in synchrony with the adult), and therefore showed reduced IFG activation during IPS.

The STS region is important for establishing visuo-motor correspondence, a process that compares the observed action with the planned movement, which is much needed for imitation^24,65^. It is more active during imitation compared to action execution and observation across different tasks, including actions involving simple finger movements^25^, and pantomimed actions on objects^24^. Children with ASD have decreased STS activation when observing biological motions and during action imitation^66,67^. Similarly, we found reduced STS activation in children with ASD during sway synchrony, suggesting that they have difficulties matching the observed actions with their own movement repertoire. Through this study, we extend our previous neurobiomarkers during reaching synchrony^13^ to whole-body sway synchrony and reconfirmed that the ASD-related neurobiomarkers are associated with IPS impairments regardless of movements performed (i.e., reaching or whole-body sway).

We found a clear increase in cortical activation when more social perceptual information (i.e., social visual and/or social tactile information) was available to the TD children. Similar results were reported in previous fMRI studies, with increased frontoparietal activation during multisensory processing^21^. The additional social visual (Face condition) and social visual plus social tactile information (Touch condition) might increase the information reaching the frontoparietal regions, leading to scaling up of cortical activation. However, children with ASD might have difficulties perceiving/processing multiple social inputs (tactile and visual) as they did not show an increase in cortical activation when more social information was available. Past studies have reported ASD-related difficulties in integrating multiple sensory (visual and tactile) and social perceptual inputs^68,69^. These difficulties in perceiving task-relevant information could affect children’s abilities to sway and engage in IPS with others.

In terms of correlations, in children with ASD, greater social functioning based on the VABS scores, greater social responsiveness based on the SRS measure, and better interpersonal skills based on the ICS measure often correlated with greater cortical activation in multiple cortical regions including the left STS, left IFG, left IPL, and right PCG regions. It is not surprising that these different measures systematically correlated with activation in these regions given their important role in social monitoring, synchrony, and perceptuo-motor aspects of interpersonal synchrony.

To our knowledge, this is the first study to investigate how different levels of social information affect whole-body IPS and cortical activation patterns in children with and without ASD. Although we identified the neurobiomarkers in children with ASD, there were some limitations to our study. The current study has a relatively small sample size which contributed to smaller effect sizes (Hedge’s g = 0.03 ~ 0.21). Although small effect sizes have often been reported in past neuroimaging studies involving children with ASD^14^, our results should be interpreted with caution. It is also important to note that the Benjamini–Hochberg method for False Discovery Rate corrections is less conservative compared to other approaches such as the Holm-Bonferroni, and may lead to more false positive results. However, Benjamini–Hochberg method is considered an acceptable trade-off between power and specificity for multichannel NIRS data as discussed in Singh & Dan (2006)^53^. With regards to sample characteristics, we did not include a cognitive measure. However, we have correlated a measure of adaptive functioning (VABS-2) and social responsiveness/impairment to behavioral and activation measures in both groups. In addition, we have few female participants in this study (n = 3), limiting our ability to identify sex-specific neurobiomarkers. Future studies should include larger samples with more female participants to further explore the ASD- and sex-specific neurobiomarkers of IPS.Using motion tracking and fNIRS systems, we identified atypical movement characteristics and associated cortical activation in children with ASD during a whole-body, sway synchrony task. Children with ASD showed hyperactivation in the IPL region during action observation and execution, hypoactivation in the IFG and STS regions during social-embedded synchronous sway, and did not show an increase in IFG and STS activation when more social information was available. Although our previous studies suggest positive behavioral outcomes in children with ASD following synchrony-based interventions^70,71^, the neural effects of such interventions are not well studied. The neurobiomarkers we identified in this study could act as objective measures of treatment response following synchrony-based interventions, perhaps leading to more normalized STS and IPL activation, post-intervention.

## Methods

### Participants

Thirty school-aged children with and without ASD participated in this study (mean age ± SE: ASD: 12.7 ± 0.8 years, 12 males and 3 females; TD: 12.5 ± 0.8 years, 10 males and 5 females; *p*s > 0.05). Participants were recruited through online announcements, phone calls and fliers distributed to local schools, ASD service and advocacy groups, as well as Simons Powering Autism Research (SPARK) participant research match service. SPARK informs their database of families about research studies in the nearby area (https://www.sfari.org/resource/spark/). Before participation, we interviewed potential participants to obtain their demographic information including age, sex, ethnicity, as well as to confirm their eligibility for participation (Table 3). Children with ASD were included if they held a professionally confirmed ASD diagnosis supported by a school record (e.g., school psychologist record confirming an ASD diagnosis) and an Individualized Education Plan (IEP) for ASD-related services or a medical or neuropsychological record from a psychiatrist or clinical psychologist using the Autism Diagnostic Observation Schedule (ADOS) and/or Autism Diagnostic Interview-Revised (ADI-R) measures. In addition, we used the Social Communication Questionnaire (SCQ)^38^ to screen for ASD symptoms. All children with ASD met criteria for a social communication delay (> 15 points) confirming the presence of a social communication difficulties, whereas all TD children scored below the cut-off score. Additionally, the two groups were matched in age and sex. Children with ASD were excluded if they had any behavioral and sensory issues that prevented them from wearing the fNIRS cap and completing the test activities. TD children were excluded if they: (a) had any neurological or developmental disorder/delays or (b) had a family history of ASD.

**Table 3:** Demographic information and the results of social communication questionnaires in ASD and TD groups.

Characteristics	ASD group (n = 15) Mean ± SE	TD group (n = 15) Mean ± SE
Age	12.7 ± 0.8	12.5 ± 0.8
Sex	12 M, 3F	10 M, 5 F
Ethnicity	1 A, 1 AC, 13 C	2 A, 13 C
**VABS-II**		
Adaptive behavior composite (%)	8.5 ± 3.6*	58.4 ± 7.3
Communication (%)	12.8 ± 4.6*	59.9 ± 6.8
Daily living (%)	18.5 ± 7.0*	58.4 ± 8.2
Socialization (%)	5.3 ± 2.1*	60.0 ± 7.9
SRS (T scores)	77.7 ± 2.7*	44.87 ± 1.62
BOT-2—body coordination (%)	16.3 ± 4.6*	72.5 ± 6.0
ICS	4.0 ± 0.3*	5.7 ± 0.2
Handedness	13 R, 2 L	14 R, 1 L
Coren’s score	31.2 ± 1.2	34.0 ± 1.6

Means and standard errors (SE) provided.

*VABS-II* Vineland Adaptive Behavior Scale-2nd Edition; *SRS* social responsiveness scale; *ICS* interpersonal communication scale; *M* male, *F* female; *C* Caucasian, *A* Asian, *AC* Asian-Caucasian. *R* right-handed, *L* left-handed. % = percentile score.

*Indicates significant differences between children with and without ASD (*p* < 0.05).

We administered the Bilateral Coordination and Balance subtests of the Bruininks-Oseretsky Test of Motor Proficiency, 2nd Edition (BOT-2) in the participating children. The children with ASD showed significantly lower Body Coordination composite percentile score (includes Bilateral coordination and Balance subtest score) compared to the TD children (*p* < 0.001; Table 3)^72^. Parents of all participating children completed the Vineland Adaptive Behavioral Scales-2nd edition (VABS-II)^39^ to assess adaptive functioning, Social Responsive Scale questionnaire (SRS)^40^ to assess social impairment, and the Interpersonal Competence Scale (ICS)^41^ to assess the child’s interpersonal social skills (Table 3). Compared to the TD children, children with ASD had significantly lower VABS-II, SRS, and ICS scores (*p*s < 0.05, Table 3). Individual data in children with ASD suggests they had well-below average to average levels of body coordination based on the BOT-2, low to moderately low levels of adaptive functioning based on the Adaptive Behavior Composite score, and moderate to severe social impairment based on the SRS T-score. Lastly, the Coren’s handedness survey was used to determine children’s handedness (Table 3)^42^. Written informed consent was obtained from parents and written/verbal assent was received from participants prior to study participation. All study procedures were carried out in accordance with the Declaration of Helsinki. All consent and assent forms as well as all study procedures were approved by the University of Delaware Institutional Review Board (UD IRB, Study Approval #: 930,721–12). Prior to study participation, written informed consent was obtained from parents who gave approval on behalf of their children as their legal guardians and written and verbal assent was obtained from the children. Written parental and experimenter permission/consent to use their pictures for this publication has been taken.

### Experimental procedures

**Figure 5:**
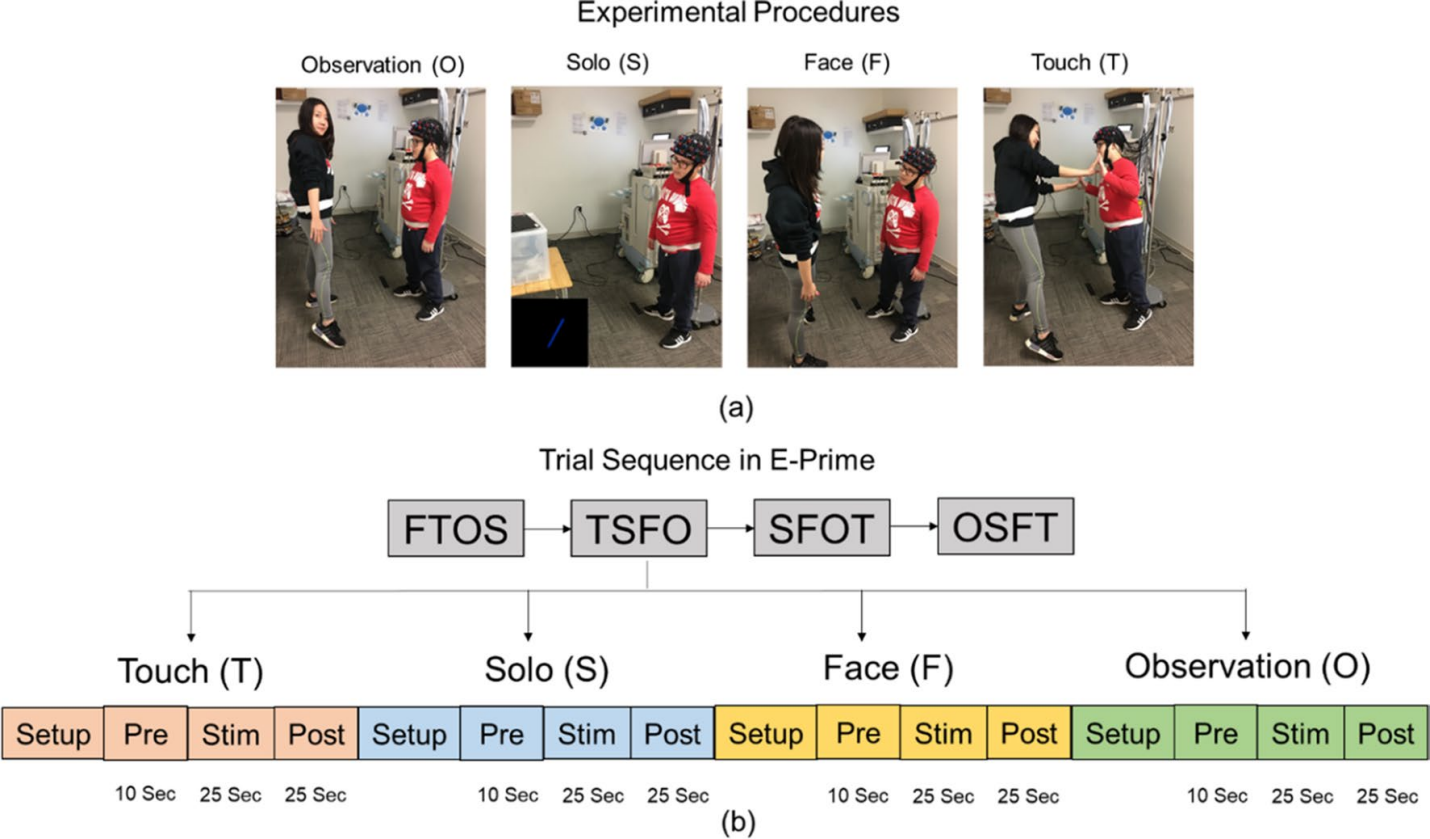
Experimental setup (**a**) and task sequence (**b**). Written parental and experimenter permission to use their pictures for this publication has been taken.

During the experiment, the children were asked to stand either facing a laptop screen or an adult tester. An fNIRS cap embedded with a 3 × 11 probe set was placed on each child’s head. Each child completed four conditions: Observation, Solo, Face, and Touch (Fig. 5a). During the *Observation* condition, the child observed an adult tester sway side to side. During the *Solo* condition, the child swayed side to side in synchrony with an oscillating bar shown on the laptop screen. During the *Face* condition, the child swayed face to face with an adult tester. Lastly, during the *Touch* condition, the child swayed face to face with an adult tester with their fingertips touching each other. The swaying speed of the tester and the oscillating bar were controlled by a rhythmic beat audible to the tester only using a wireless earphone, therefore the child had to synchronize their sway with the tester using social-visual and/or social-tactile information. The speed of the bar/tester’s motions changed within the trial, either moved faster from 40–50 bpm, or slower from 40 to 30 bpm. The children completed a total of 16 trials (4 trials per condition) that were randomized across the entire session (Fig. 5b). Each trial included a 10-s pre-stimulation, 25-s stimulation, and a 25-s post-stimulation period. The pre- and post-stimulation periods were included to account for baseline drifts in the fNIRS signal and to allow the hemodynamic response to return to baseline before starting the next trial. During baseline periods, the children were asked to stand still and focus on a crosshair on the front wall.

### Data collection

We used a 52-channel Hitachi fNIRS system (ETG-4000, Hitachi Medical Systems, Inc., sampling rate: 10 Hz) to record the hemodynamic changes over the regions of interest (ROIs). A 3 × 11 probe set consisting of 17 infrared emitters and 16 receivers was positioned over bilateral frontoparietal and temporal regions. The middle column of the probe set was aligned with the child’s nasion while the bottom row was aligned with the child’s eyebrow (See Supplementary Fig. S1a online). Each adjacent pair of probes are located 3 cm apart on the cap and act as an emitter and a receiver of two wavelengths of infrared light (695 and 830 nm). Infrared light passes through the skull creating a banana-shaped arc and reaches the cortical area approximately below the midpoint of the two probes forming a channel. The attenuation of infrared light was used to calculate the changes in concentrations of oxygenated (HbO_2_) and deoxygenated hemoglobin chromophores (HHb) per channel using the Modified Beer-Lambert Law^37^. An increase in the concentration of HbO_2_ and a decrease in HHb concentration were expected when a certain region underneath a channel was active^37^. Additionally, inertial measurement units (IMUs, Xsens, Inc.) were placed on the mid-point of the second sacral vertebrae (i.e., body’s center of mass) for the child and the tester to record body displacements during swaying (Sampling rate: 100 Hz; see Supplementary Fig. S1c online). The Hitachi fNIRS system and the IMU systems were synchronized and triggered by E-Prime presentation software (version 2.0, https://pstnet.com/). The entire session was videotaped using a camcorder that was also synchronized with the fNIRS system.

### Spatial registration approach

We used the ETG-4000 3D positioning unit, a reference coordinate system, to conduct 3D registration. During registration, we recorded the 3D locations of the standard cranial landmarks (i.e., nasion, inion, tragus points of the ears, and the Cz position of the International 10–20 system) and each of the probes. The 3D coordinates of all participating children were run through MATLAB codes developed by our collaborator/co-author Dr. Tsuzuki^43^ to estimate and label the channel locations using the LONI Probabilistic Brain Atlas (LPBA)^44^. For further details, readers may refer to our earlier publications^13,45,46^. A channel was included if more than 60% of a channel (i.e., each channel was as the centroid of sphere) was within a given ROI and was excluded if it was not. A channel was also excluded if its homologue belonged to another ROI (e.g., the homologous channels 12 and 20 were excluded because channel 12 was assigned to IPL region while channel 20 was assigned to the PCG region). Based on these rules, we assigned 32 out of the 52 channels to five ROIs in each hemisphere (See Supplementary Fig. S1b and Table S3 online): (i) MFG (left: channels 7, 8, 17, 18, 28, 38; right: channels 3, 4, 14, 15, 25, 36); (ii) IFG (left: channels 29, 39, 50; right: channels 24, 35, 45); (iii) PCG (left: channels 9, 30; right: channels 2, 23); (iv) STS (left: channels 42, 51, 52; right: channels 32, 43, 44); and (v) IPL (left: channels 10, 21; right: channels 1, 11).

### Data processing

Data on displacement of the body’s center of mass were obtained from the child and the adult and were later analyzed using custom MATLAB codes. First, we low-pass filtered the displacement data using a 4^th^ order, zero-lag Butterworth filter using a cut-off frequency of 10 Hz. Then, we extracted the amplitudes in mm for each child. To calculate IPS, we used Cross-Spectral Analysis (CSA)^47^ to determine the level of coherence between the child and the tester. Coherence is a measure of the degree of correlation between the signals within a certain frequency and it ranges from 0 to 1 (i.e., 0 presents no synchrony and 1 presents perfect synchrony). The CSA approach had been used in previous studies to analyze correlations between rhythmic sway data between two individuals^48^.

To analyze the fNIRS data, we incorporated open-source software—Hitachi POTATo^49^ and Homer-2^50^—within custom MATLAB codes. Specifically, we band-pass filtered the data between 0.01 and 0.5 Hz to remove high- and low-frequency noise, used the wavelet method to remove movement artifacts, used General Linear Modeling (GLM) to estimate the hemodynamic response function, and corrected for baseline drifts by subtracting the linear trend between the pre- and post-stimulation baselines from values in the stimulation period^49,50^. Lastly, we averaged the HbO_2_ and HHb values over each stimulation period for each trial. The HbO_2_ data has a greater range and signal to noise ratio compared to the HHb data and is more often reported in the literature^51^. The data were plotted and saved at each step for visual screening. For additional details, readers may refer to our earlier publications^13,45,46^.

### Data exclusion

Cortical activation and movement kinematic data were excluded based on visual screening of video and MATLAB figures. A trained student researcher scored the session videos to exclude trials with significant behavioral errors (e.g., child did not follow task instructions or spoke to the tester during the stimulation period). This criterion resulted in data exclusion of 5% from the ASD group and 1% from the TD group. In addition, we screened the MATLAB figures of cortical activation data at each step to exclude channels with no signals (i.e., appeared as flat lines due to bad probe-scalp contact). 12% of data from the ASD and 5% of data from the TD groups were removed based on channel checking. In total, 15% data from the ASD group and 6% data from the TD group and were excluded and 85–94% of data were retained in our analysis.

### Statistical analyses

For sway amplitude and coherence data from the IMU system, t-tests were used to compare group/condition-related differences. For the cortical activation data, we averaged data across channels within the same ROI based on our spatial registration output (See Supplementary Fig. S1b online) to avoid multiple comparisons. We determined levels of activation for 10 ROIs, including the MFG, IFG, PCG, STS, and IPL regions for both left and right hemispheres. Using IBM SPSS (SPSS, Inc.), a repeated-measures ANOVA was conducted using within-subject factors of condition (Observation, Solo, Face, Touch), hemisphere (left, right), and ROI (MFG, IFG, PCG, STS, IPL) and a between-subjects factor of group (TD, ASD) for average HbO_2_ values. Since we found group differences in movement amplitude and BOT-2 Body Coordination scores, the two variables were added as covariates to control for cortical activation associated with differences in movement characteristics. Greenhouse–Geisser corrections were applied when our data violated the sphericity assumption based on Mauchly’s test of sphericity. Differences in social communication skills are associated with the ASD diagnosis and ideally should not be controlled by treating them as covariates^52^, therefore, we used Pearson’s correlations to investigate the relationships between cortical activation, movement characteristics, and social communication performance assessed by three measures—VABS, SRS, and ICS.

To account for multiple comparisons (post hoc t-tests following ANOVA runs and correlational analyses), we used the False Discovery Rate (FDR) method to adjust the cut-off for statistical significance^53^. Specifically, the Benjamini–Hochberg method was used wherein unadjusted *p*-values were rank ordered from low to high. Statistical significance was declared if the unadjusted *p*-value was less than the *p*-value threshold. *p*-value threshold was determined by multiplying 0.05 with the ratio of the unadjusted *p*-value rank to the total number of comparisons (*p*-threshold for ith comparison = 0.05 × i/n; where n = total number of comparisons). We also report effect sizes for significant post-hoc comparisons using the Hedge’s g method^73^.

## Supplementary Information

Supplementary Information.

## Data Availability

The datasets generated during and/or analysed during the current study are available from the corresponding author on reasonable request.
