# Supplementary material for: Movement kinematics and cortical activation in children with and without autism spectrum disorder during sway synchrony tasks: an fNIRS study
Source: Sci Rep. 2021 Jul 22;11:15035. doi: 10.1038/s41598-021-94519-4 (PMC8298433; doi:10.1038/s41598-021-94519-4)

Movement Kinematics and Cortical Activation in Children with and without Autism Spectrum Disorder During Sway Synchrony Tasks: An fNIRS Study

Wan-Chun Su,[1,2] McKenzie Culotta,[1,2] Daisuke Tsuzuki,[3] Anjana Bhat,[1,2,4*]

Supplementary Table S1. Means and standard errors of HbO_2 concentration in children with and without ASD during Observation, Solo, Face, and Touch conditions of the sway synchrony task.

**Group activation data**	**Observation**		**Solo**		**Face**		**Touch**	
	**Mean**	**SE**	**Mean**	**SE**	**Mean**	**SE**	**Mean**	**SE**
**TD**								
Left hemisphere								
MFG	0.017	0.009	0.019	0.008	0.011	0.009	0.021	0.009
IFG	0.023	0.011	0.031	0.009	0.035	0.010	0.053	0.010
PCG	0.013	0.010	0.035	0.010	0.037	0.012	0.043	0.010
STS	0.031	0.015	0.089	0.014	0.103	0.017	0.115	0.015
IPL	0.020	0.010	0.018	0.013	0.032	0.013	0.035	0.010
Right hemisphere								
MFG	0.013	0.008	0.026	0.009	0.022	0.009	0.028	0.009
IFG	0.033	0.012	0.047	0.011	0.060	0.012	0.072	0.010
PCG	0.013	0.009	0.033	0.011	0.061	0.013	0.050	0.008
STS	-0.007	0.010	0.074	0.012	0.093	0.011	0.100	0.013
IPL	0.008	0.008	0.008	0.010	0.007	0.009	0.022	0.010
**ASD**								
Left hemisphere								
MFG	0.012	0.006	0.015	0.007	0.015	0.007	0.013	0.007
IFG	0.028	0.009	0.029	0.010	0.050	0.010	0.034	0.008
PCG	0.027	0.009	0.064	0.010	0.069	0.009	0.063	0.011
STS	0.033	0.009	0.053	0.011	0.063	0.011	0.062	0.010
IPL	0.037	0.009	0.052	0.011	0.043	0.010	0.040	0.010
Right hemisphere								
MFG	0.016	0.007	0.016	0.007	0.013	0.009	0.015	0.009
IFG	0.044	0.011	0.024	0.009	0.039	0.011	0.035	0.011
PCG	-0.005	0.010	0.033	0.010	0.012	0.010	0.008	0.010
STS	0.005	0.010	0.053	0.012	0.056	0.012	0.069	0.012
IPL	0.045	0.013	0.045	0.009	0.038	0.010	0.039	0.008

Supplementary Table S2. Post-hoc analyses for 3-way interactions of Group × Condition × Region.

**Comparison**	***p*-values**	**Direction of effect**
**Group related difference**		
Observation, IPL	0.008	ASD > TD[a]
Solo, STS	0.021	TD > ASD[b]
Solo, IPL	0.001	ASD > TD[a]
Face, STS	0.004	TD > ASD[a]
Together, IFG	0.005	TD > ASD[a]
Together, STS	0.001	TD > ASD[a]
**Condition related difference**		
TD, IFG	0.024	Face > Observation[b]
	< 0.001	Touch > Observation[a]
	0.001	Touch > Solo[a]
	0.021	Touch > Face[b]
TD, PCG	0.026	Solo > Observation[b]
	< 0.001	Face > Observation[a]
	< 0.001	Touch > Observation[a]
TD, STS	< 0.001	Solo > Observation[a]
	< 0.001	Face > Observation[a]
	< 0.001	Touch > Observation[a]
	0.024	Touch > Solo[b]
ASD, PCG	< 0.001	Solo > Observation[a]
	0.002	Face > Observation[a]
	0.011	Touch > Observation[b]
ASD, STS	0.001	Solo > Observation[a]
	< 0.001	Face > Observation[a]
	< 0.001	Touch > Observation[a]

[a]*p*-values which survived the FDR correction. [b]*p*-values < 0.05 which did not survive FDR correction.

Supplementary Table S3. Channel assignments based on the anchor registration approach. For each channel, the spatial location in the MNI coordinate system and the probability of covering different brain regions are shown. The channels are symmetrically divided across the two hemispheres (left, right). The color-coded channels were considered within a specific ROI (MFG, IFG, PCG, STS, and IPL).

Side	CH	MNI's coordinate			MFG	IFG	PCG		STS		IPL		Other		Assigned region
		X	Y	Z	Middle frontal gyrus	Inferior frontal gyrus	Precentral gyrus	Postcentral gyrus	Superior temporal gyrus	Middle temporal gyrus	Supramarginal gyrus	Angular gyrus	Superior frontal gyrus	orbitofrontal gyrus	
Left	7	-30.7	43.7	42.7	88.1								11.9		MFG
	8	-47.7	23.7	44.7	100.0										MFG
	9	-58.0	-2.7	45.3			72.9	27.1							PCG
	10	-63.0	-28.7	47.3				9.0			91.0				IPL
	17	-22.7	59.7	31.7	96.4								3.6		MFG
	18	-44.3	41.3	31.3	100.0										MFG
	19	-56.3	15.3	31.7	37.2	23.8	39.0								Excluded
	20	-66.0	-13.3	34.7				65.3			34.7				Excluded
	21	-66.0	-40.7	38.3							91.1	8.9			IPL
	28	-37.3	58.3	20.3	100.0										MFG
	29	-54.0	33.7	19.7	30.3	69.7									IFG
	30	-65.0	3.3	21.7		1.6	72.0	26.3							PCG
	31	-69.0	-26.6	25.3				7.5	28.0		64.4				Excluded
	38	-24.3	70.0	6.3	99.0								1.0		MFG
	39	-47.7	50.3	5.7	16.4	83.6									IFG
	40	-58.7	22.7	7.7	100.0										Excluded
	41	-67.0	-10.3	9.3			1.3	49.7	49.0						Excluded
	42	-70.0	-39.3	11.7					80.1	19.9					STS
	49	-37.0	63.0	-7.0	42.4	28.6								29.0	Excluded
	50	-53.0	40.7	-6.7		56.4								43.6	IFG
	51	-62.0	5.7	-6.3		4.2	1.8		87.7	6.3					STS
	52	-71.0	-23.3	-5.3					6.9	93.1					STS
Center	5	17.3	55.3	42.3	60.1								39.9		Excluded
	6	-8.3	56.3	43.7	6.0								94.0		Excluded
	16	6.3	65.0	30.7	22.4								71.6		Excluded
	26	19.7	70.0	19.7	94.5								5.5		Excluded
	27	-11.3	70.0	20.3	25.4								74.6		Excluded
	37	5.3	72.3	5.7	29.8								70.2		Excluded
	47	19.3	71.7	-7.7	75.9								5.3	18.8	Excluded
	48	-10.3	72.0	-7.3	11.3								85.6	3.0	Excluded
Right	1	67.0	-34.7	43.7							93.0	7.0			IPL
	2	64.0	-7.7	42.3			12.4	55.5			32.2				PCG
	3	53.0	19.7	41.3	71.6	5.4	23.0								MFG
	4	38.7	41.7	41.7	100.0										MFG
	11	67.0	-45.3	34.3					5.6		36.1	58.3			IPL

	12	69.7	-18.3	31.3				24.8	1.3		73.9				Excluded
	13	63.7	10.7	29.3		4.0	92.4	3.6							Excluded
	14	51.0	38.3	39.3	66.7	33.3									MFG
	15	30.3	58.3	29.3	100.0										MFG
	22	71	-31.7	20.7					54.8		34.7	10.5			Excluded
	23	68.7	-0.7	18.7			21.7	77.0			1.3				PCG
	24	59.3	30.3	17.3		90.7	9.3								IFG
	25	44.3	55.7	17.7	64.9	35.1									MFG
	32	71.0	-44.3	7.3					18.6	81.4					STS
	33	72.0	-15.3	4.3				1.3	85.3	13.5					Excluded
	34	61.3	17.3	6.3		37.0	52.6		10.4						Excluded
	35	53.0	46.3	4.3	3.9	96.1									IFG
	36	33.3	67.0	4.7	93.8	6.3									MFG
	43	73.0	-28.3	-8.3						100.0					STS
	44	67.0	0.3	-10.3					40.9	59.1					STS
	45	56.0	35.7	-7.7		73.1								26.9	IFG
	46	43.7	59.7	-7.7	2.9	66.8								30.3	Excluded

Supplementary Figure S1. Probe placement (a), spatial registration output (b), and IMU system placement (c). Written permission has been taken for publication of participant pictures. MFG = middle frontal gyrus, IFG = inferior frontal gyrus; PCG = pre/post central gyrus; STS = superior temporal sulcus; IPL = inferior parietal lobe.

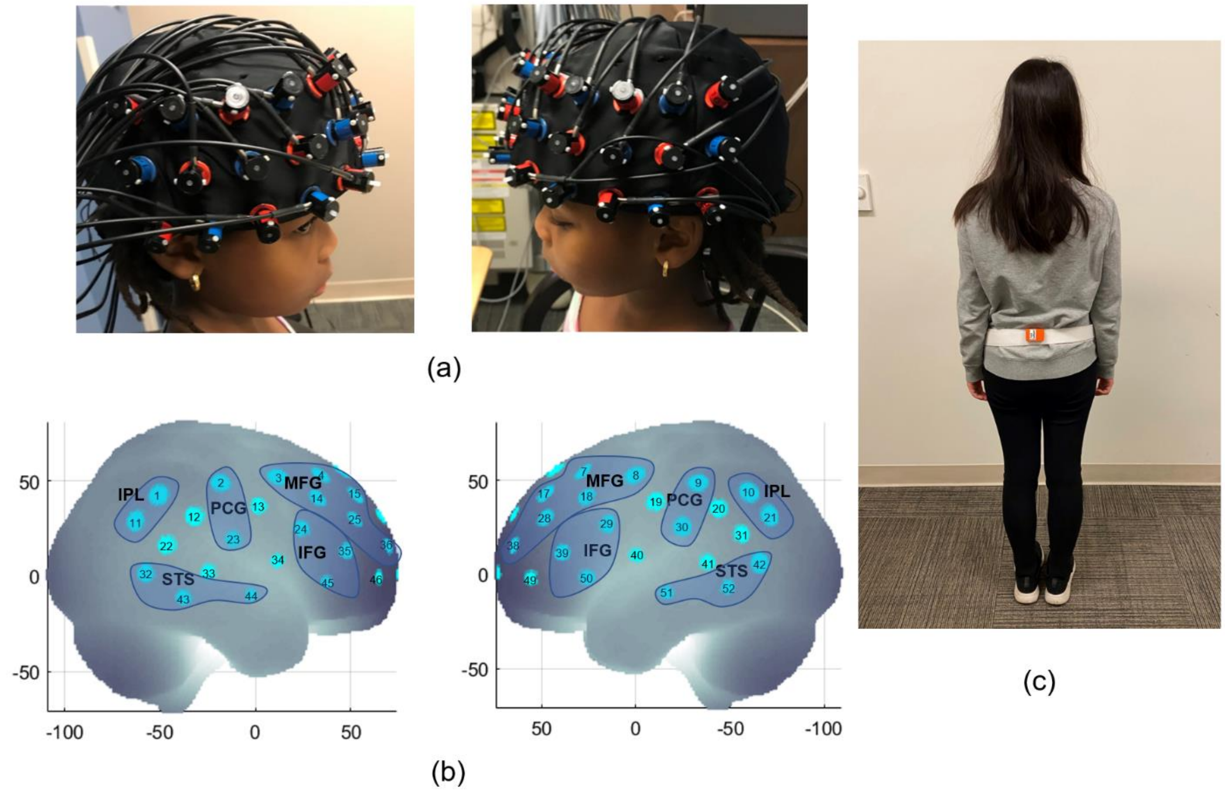

Supplement: Supplementary file 1 — Supplementary Information. [file 41598_2021_94519_MOESM1_ESM.pdf]
